# Relationship between atrial septal defects and asthma-like dyspnoea: the impact of transcatheter closure

**DOI:** 10.1007/s12471-016-0879-6

**Published:** 2016-08-25

**Authors:** M. Nassif, C. B. B. C. Heuschen, H. Lu, B. J. Bouma, R. P. van Steenwijk, P. J. Sterk, B. J. M. Mulder, R. J. de Winter

**Affiliations:** 1 Department of Cardiology, Academic Medical Center – University of Amsterdam, Amsterdam, The Netherlands; 2 Department of Pulmonary Medicine, Academic Medical Center – University of Amsterdam, Amsterdam, The Netherlands; 3 Interuniversity Cardiology Institute of the Netherlands (ICIN), Utrecht, The Netherlands

**Keywords:** Heart septal defects, Atrial Dyspnoea, Bronchial hyperreactivity, Asthma, Device closure

## Abstract

**Background:**

Patients with atrial septal defects (ASD) are often misdiagnosed as asthma patients and accordingly receive erroneous bronchodilator treatment. In order to characterise their symptoms of dyspnoea to explain this clinical observation, we investigated the prevalence of asthma-like symptoms in patients with secundum ASD who then underwent successful percutaneous closure.

**Methods:**

A total of 80 ASD patients (74 % female, mean age 46.7 ± 16.8 years, median follow-up 3.0 [2.0–5.0] years) retrospectively completed dyspnoea questionnaires determining the presence and extent of cough, wheezing, chest tightness, effort dyspnoea and bronchodilator use on a 7-point scale (0 = none, 6 = maximum) before and after ASD closure. The Mini Asthma Quality of Life (Mini-AQLQ) and Asthma Control Questionnaire with bronchodilator use (ACQ6) were administered.

**Results:**

A total of 48 (60 %) patients reported cough, 27 (34 %) wheezing, 26 (33 %) chest tightness and 62 (78 %) effort dyspnoea. Symptom resolution or reduction was found in 64 (80 %) patients after ASD closure. Asthma symptom scores decreased significantly on the Mini-AQLQ and ACQ6 (both *p* < 0.001). The number of patients using bronchodilators decreased from 16 (20 %) to 8 (10 %) patients after ASD closure (*p* = 0.039) with less frequent use of bronchodilators (*p* = 0.015).

**Conclusions:**

A high prevalence of asthma-like symptoms and bronchodilator use is present in ASD patients, which exceeds the low prevalence of bronchial asthma in this study population. Future prospective research is required to confirm this phenomenon. The presence of an ASD should be considered in the differential diagnosis of patients with asthma-like symptoms, after which significant symptom relief can be achieved by ASD closure.

## Introduction

The ostium secundum atrial septal defect (ASD) is one of the most commonly diagnosed congenital heart diseases among adults. Patients with ASD usually present with symptoms of dyspnoea, which may develop with increasing age. Due to the epidemiologically more prevalent asthma in the general population, we observed that dyspnoeic ASD patients are often erroneously diagnosed with asthma and receive lengthy bronchodilator treatment. Delay in the correct diagnosis and treatment of ASD leads to complications of long-standing right ventricular volume and eventually pressure overload such as atrial arrhythmias, paradoxical embolism, pulmonary hypertension and right ventricular failure [1, 2].

Percutaneous ASD closure is the treatment of choice in haemodynamically significant left-to-right shunts with suitable anatomy [3]. Previous studies have already shown dyspnoea to decrease after percutaneous closure, and several have indeed found objective pulmonary function improvement by cardiopulmonary exercise testing [4–7]. However, to date the characteristics of dyspnoea reported by these ASD patients have not been investigated.

The purpose of this study was to examine the prevalence of asthma-like symptoms in patients with ASD, and investigate whether percutaneous closure of the left-to-right shunt affects asthma-like symptoms both in diagnosed asthma patients and in non-asthmatics.

## Methods

### Patient population

Between 2005 and 2013 a total of 105 consecutive adult patients underwent percutaneous closure of an ASD in our centre using the Amplatzer Septal Occluder device (AGA Medical, Minneapolis, Minnesota, USA). Of the 89 patients with successful percutaneous closure of their ASD (16 converted to surgery), 80 patients were available for follow-up and were therefore included in this study. Follow-up was fully obtained (*n* = 80). This study was conducted in accordance with all human research regulatory guidelines and the need to obtain informed consent was waived by the institutional ethics committee.

### Procedure

Percutaneous ASD closure was performed under general anaesthesia for transoesophageal echocardiography guidance to ensure optimal device placement. Heparin and aspirin were routinely administered at the start of the procedure. Successful ASD closure was defined as correct device position without post-procedural complications.

### Follow-up evaluation

Patients were discharged one day post-procedurally after confirming a complete ASD closure on transthoracic echocardiography (TTE). Aspirin (100 mg daily) and clopidogrel (600 mg loading dose and 75 mg daily) were prescribed for six months along with standard endocarditis prophylaxis. Patients were followed clinically and TTE was performed at least one day and six months after device implantation.

### Definition of outcomes

Primary outcomes were the prevalence of asthma-like symptoms, defined as wheeze, chest tightness, cough, effort dyspnoea and bronchodilator use, before and after successful percutaneous ASD closure. Secondary outcome measures were the Mini Asthma Quality of Life Questionnaire symptom and environmental score (Mini-AQLQ, minimum clinically significant difference =0.5) and asthma control level (Asthma Control Questionnaire with bronchodilator use, ACQ6) [8–10]. The extent of asthma-like symptoms was quantified using the same score range as the above-mentioned validated questionnaires (range 0–6, lower score indicating less symptoms) as conducted by telephone contact.

The primary and secondary outcomes are defined in the overall group, as well as in the subgroups of asthmatics and non-asthmatics. Asthmatics are defined as patients with a physician’s diagnosis of bronchial asthma by presence of variable expiratory airflow obstruction. Conveniently, symptoms of wheezing, chest tightness, cough and effort dyspnoea are referred to as asthma-like symptoms, even if reported by patients with bronchial asthma.

### Statistical analysis

The Mini-AQLQ score was inverted for data analyses to be consistent with a lower score indicating less severity. Dichotomous variables were analysed using the McNemar test and are expressed as frequency (percentage). The continuous, non-parametric variables were analysed using the Wilcoxon signed-rank test (baseline vs. follow-up) and the Mann-Whitney-U test (asthmatics vs. non-asthmatics) and are expressed as median (25^th^–75^th^ percentile). A *p* value <0.05 was considered statistically significant. All statistical analyses were made using IBM SPSS Statistics for Windows, Version 21 (IBM Corp., Armonk, NY, USA).

## Results

### ASD closure

Baseline characteristics of the 80 patients with successful percutaneous closure are shown in Table 1. TTE follow-up at six to nine months post-procedurally was available in all patients and showed a trivial residual shunt in six patients (8 %). No device- or procedure-related complications occurred during clinical follow-up.

**Table 1:** Baseline characteristics of unrepaired ASD patients

	*n* = 80	
**Demographics**		
Age (years)	46	±16.8
Sex (male)	21	(26 %)
Body mass index (kg/m²)	25.6	±4.6
Former smoker	14	(18 %)
Current smoker	17	(21 %)
**Clinical history**		
COPD^a^	2	(3 %)
Bronchial asthma^a^	6	(8 %)
Atopic constitution^b^	14	(18 %)
Pulmonary hypertension^c^	5	(6 %)
Myocardial infarction	0	(0 %)
Acute heart failure	7	(9 %)
– Right ventricular	5	(6 %)
Arrhythmia	19	(24 %)
**ASD-related characteristics** ^d^		
SPAP (mm Hg)	35	[30–41]
RVEDV (ml)	239	±117
Qp:Qs ratio	1.8	[1.4–2.0]
Defect size (mm)	17.7	±7.5
Device size (mm)	21.8	±7.4
Closure indication		
– Right ventricular overload	71	(89 %)
– Paradoxical embolism	9	(11 %)

Values are in numbers (%), mean ± SD or median [25th–75th percentile]. *COPD* chronic obstructive pulmonary disease; *SPAP* systolic pulmonary artery pressure, normally ≤36 mm Hg; *RVEDV* right ventricular end-diastolic volume, normal range 100–160 ml; *Qp* Qs ratio pulmonary to systemic shunt fraction

^a^physician’s diagnosis by pulmonary function testing; ^b^atopic dermatitis, allergic rhinitis and/or bronchial asthma; ^c^defined as right ventricular systolic pressure ≥50 mm Hg on echocardiography; ^d^measured on echocardiography

### Asthma-like symptoms

Of all 80 patients with unrepaired ASD, 48 reported cough (60 %), 27 wheezing (34 %), 26 chest tightness (33 %) and 62 effort dyspnoea (78 %). After successful percutaneous closure, 64 (80 %) reported either complete resolution or symptom reduction (*p* < 0.001) at a median follow-up of 3.0 (2.0–5.0) years.

**Fig. 1:**
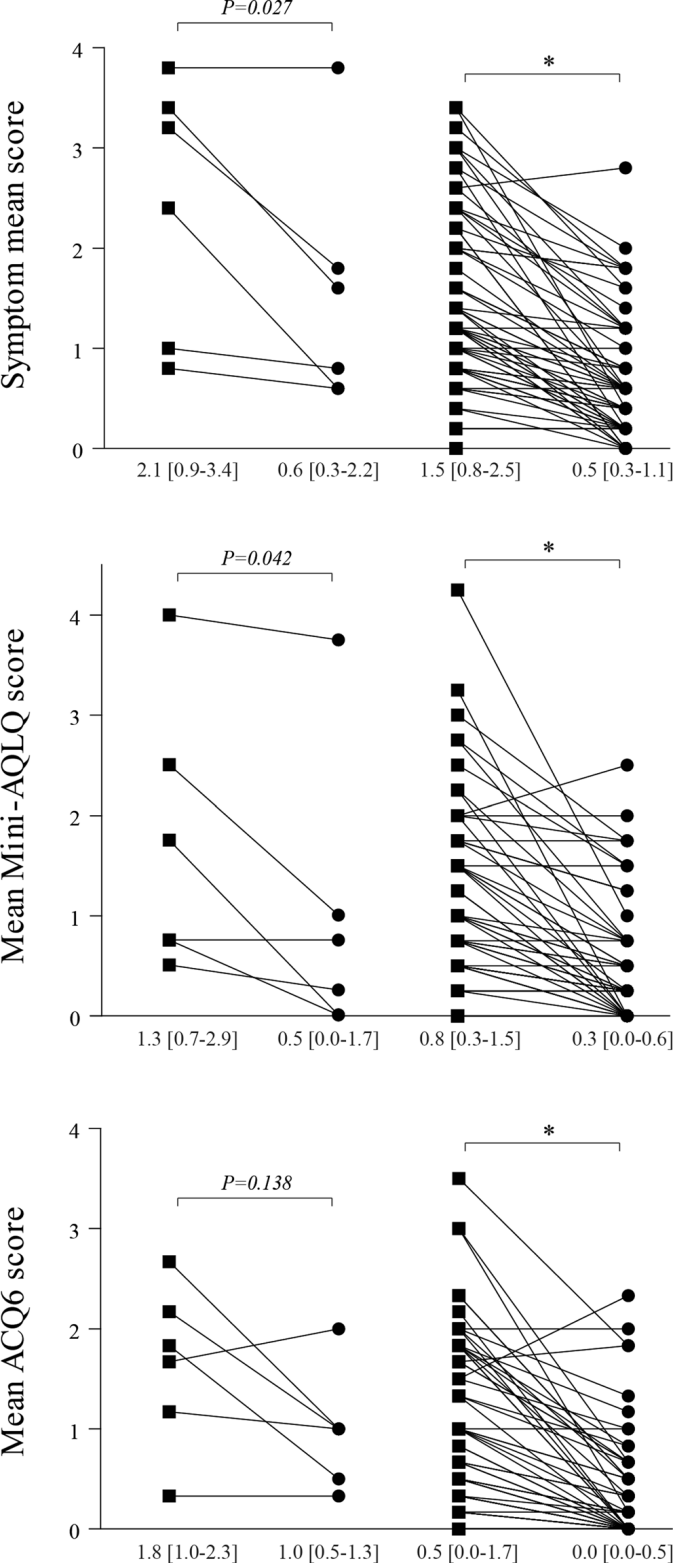
Symptom severity and scores of the Mini-AQLQ and ACQ at baseline (square) and follow-up (circle) in asthmatics (left, *n* = 6) and non-asthmatics (right, *n* = 74) separately. Values are given in median [25^th^–75^th^ percentile]. Score 0 = none, 1 = a very little, 2 = a little, 3 = a moderate amount, 4 = quite a lot, 5 = a great deal, 6 = a very great deal. *p < 0.001

Table 2 and 3 show the quantified effect of ASD closure on asthma-like symptoms and in the Mini-AQLQ (symptom and environment domain) and ACQ6 (bronchodilator use separately and within the overall ACQ6 score). Symptom severity significantly decreased from baseline to follow-up in all questionnaire scores. This effect was found in both asthmatics and non-asthmatics, although in the latter a larger change was observed (Fig. 1).

**Table 2:** Prevalence of asthma-like symptoms in ASD patients with and without asthma and the impact of transcatheter closure

	All patients Values are in numbers (%)		Non-asthmatics Values are in numbers (%)		Asthmatics Values are in numbers (%)	
	*n* = 80		*n* = 74		*n*=6	
**Baseline symptom prevalence**						
Cough	48	(60)	43	(58)	5	(83)
Wheezing	27	(34)	21	(28)	6	(100)
Chest tightness	26	(33)	23	(31)	3	(50)
Effort dyspnoea	62	(78)	57	(77)	5	(83)
None	10	(13)	10	(14)	0	(0)
**Symptom resolution**						
Cough	8/48	(17)*	6/43	(14)	2/5	(40)
Wheezing	14/27	(52)**	12/21	(57)	2/6	(33)
Chest tightness	13/26	(50)***	11/23	(48)	2/3	(67)
Effort dyspnoea	14/62	(23)***	13/57	(23)	1/5	(20)
**Symptom reduction**						
Cough	19/48	(40)***	17/43	(40)	2/5	(40)
Wheezing	5/27	(19)***	3/21	(14)	2/6	(33)
Chest tightness	7/26	(27)***	7/23	(30)	0/3	(0)
Effort dyspnoea	38/62	(61)***	34/57	(60)	4/5	(80)
**Overall symptom score change**						
Resolution or reduction	64/80	(80)***	58/74	(78)	6/6	(100)
Any symptom	30/80	(38)	28/74	(38)	2/6	(33)
≥2 symptoms	34/80	(43)	30/74	(41)	4/6	(67)
Equal	16/80	(20)^a^	15/74	(20)	1/6	(17)
Worsening	1/80	(1)	1/74	(1)	0/6	(0)

Symptom reduction and score change is defined as a ≥ 1 grade change on a 7-point scale; percentages may not sum to 100 % due to rounding; *ASD* atrial septal defect; *p < 0.05. **p = 0.001; ***p < 0.001 in relation with baseline incidence of symptoms

^a^includes all ten asymptomatic patients at baseline

**Table 3:** Quantification of symptom improvement and bronchodilator use at baseline and follow-up

	*n*	Baseline		Follow-up		*p* value
**Symptom score**						
Cough	48	2.00	[1.00–4.00]	1.00	[1.00–2.00]	*p* < 0.001
Wheezing	27	2.00	[1.00–2.00]	0.00	[0.00–1.00]	*p* < 0.001
Chest tightness	26	2.00	[2.00–3.00]	0.50	[0.00–2.00]	*p* < 0.001
Effort dyspnoea	62	5.00	[3.00–6.00]	2.00	[1.00–3.00]	*p* < 0.001
Symptom mean score	70	1.50	[0.81–2.50]	0.50	[0.25–1.19]	*p* < 0.001
**Mini-AQLQ**						
Symptom domain score	80	0.75	[0.25–1.69]	0.25	[0.00–0.75]	*p* < 0.001
Environment domain score	80	0.00	[0.00–1.00]	0.00	[0.00–0.67]	*p* = 0.006
**ACQ6**						
ACQ6 mean score	80	0.67	[0.00–1.67]	0.00	[0.00–0.50]	*p* < 0.001
Bronchodilator use	80	16	(20 %)	8	(10 %)	*p* = 0.039
Score bronchodilator use	16	3.00	[1.50–3.00]	0.00	[0.00–2.50]	*p* = 0.015

Values are in median [25^th^–75^th^ percentile] or in numbers (percentages); all given scores are presented as the questionnaire mean score, range 0 = minimum and 6 = maximum

*Mini-AQLQ* Mini Asthma Quality of Life Questionnaire; *ACQ6* Asthma Control Questionnaire with bronchodilator use

### Bronchodilator use

**Fig. 2:**
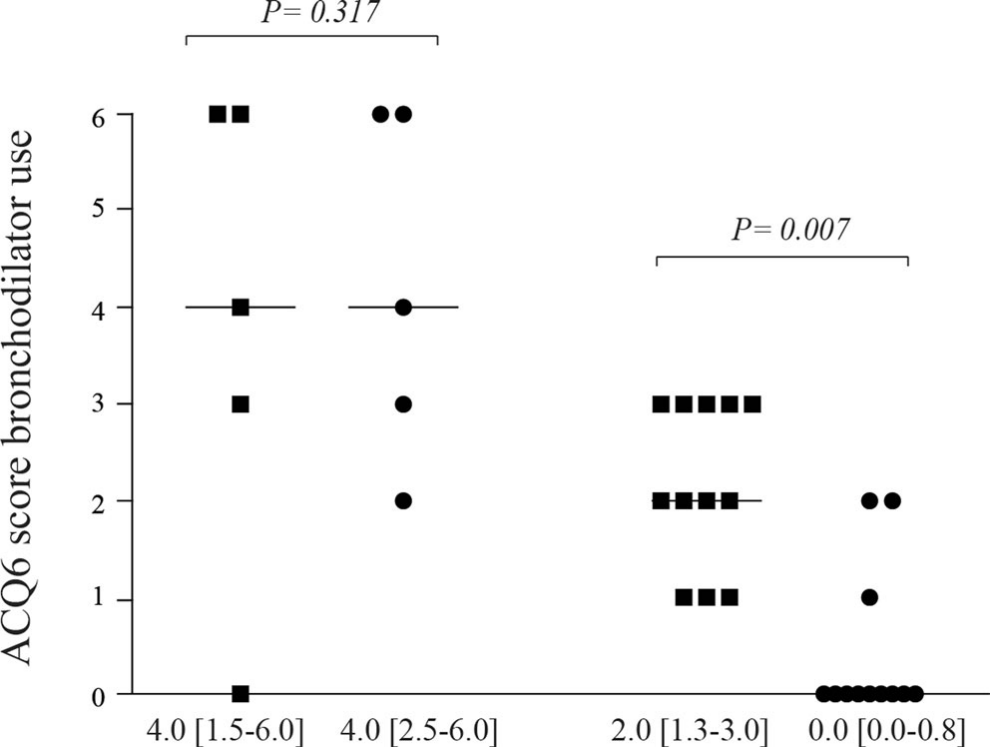
The frequency of bronchodilator use as assessed by the ACQ6 in patients with current bronchodilator use (*n* = 16) at baseline (square) and follow-up (circle) separated as asthmatics (*left*) versus non-asthmatics (*right*). Values are given in median [25^th^–75^th^ percentile]. Score 0 = none, 1 = 1–2 puffs most days, 2 = 3–4 puffs most days, 3 = 5–8 puffs most days, 4 = 9–12 puffs most days, 5 = 13–16 puffs most days, 6 = more than 16 puffs most days

Of all 80 patients, 16 patients (20 %) reported current bronchodilator use, which decreased to eight patients (10 %) after closure (*p* = 0.039). The prevalence and frequency of use is shown in Fig. 2. In contrast with the significantly reduced frequency in non-asthmatics, asthmatic ASD patients continued to use bronchodilators and in the same frequency as before closure. One asthmatic patient had no current bronchodilator use at baseline but started using them in the period after closure, making up a total of eight patients (10 %) with current bronchodilator use at follow-up.

## Discussion

The results of our observational study show a high prevalence of asthma-like symptoms in yet unrepaired ASD patients: 70 of 80 patients (88 %) reported cough, wheezing, chest tightness and/or effort dyspnoea. This cannot be accounted for by the low prevalence of either the number of diagnosed asthma patients in this study population (8 %) or the prevalence of bronchial hyperreactivity in the general Dutch population (maximum 25 % including asymptomatic patients) [11]. Percutaneous ASD closure results in symptom resolution or significant reduction (≥1 grade on a 7-point scale) in the majority of patients (80 %, *p* < 0.001), both in non-asthmatics and asthmatics alike.

Bronchial asthma is characterised by variable airflow limitation and by airway hyperreactivity, which represents an exaggerated contractile response of the airway smooth muscle to various stimuli [12]. Atopy is the strongest identifiable risk factor for the development of asthma. Pulmonary congestion by congenital heart disease has been suggested to increase the risk of atopic asthma in genetically predisposed children, and most reports of coexisting asthma in congenital heart disease attributed this to the presence of pulmonary hypertension [13–15]. In our patient population, however, only a small percentage of atopic constitution (18 %) and a very low Mini-AQLQ environment domain score (median 0.00 [0.00–1.00]) was present. Similarly, pulmonary hypertension was diagnosed using echocardiography in only five patients (6 %), albeit in the absence of dynamic stress echocardiography to potentially detect latent pulmonary hypertension [16, 17]. Furthermore, in this study ASD patients with diagnosed bronchial asthma showed symptom reduction similar to the non-asthmatics. The reported asthma-like symptoms are therefore unlikely fully the result of the bronchial asthma itself, but rather due to a related mechanism common in all ASD patients.

Alternatively, the left-to-right shunt in ASD patients might contribute to any of the inflammatory, physiological, and structural factors in the pathogenesis of asthma or in the initiation of bronchial hyperreactivity. Several experimental studies have suggested an association between bronchial hyperreactivity and impaired left ventricular function, chronic elevation in left atrial pressure, and precapillary pulmonary hypertension due to aorto-caval shunts [18–20]. In clinical studies, bronchial hyperreactivity has been reported in patients with lung congestion secondary to mitral valve disease, ischaemic heart disease, and chronic heart failure [21–24]. The proposed mechanisms are interstitial and/or airway wall oedema, bronchial wall muscle hypertrophy [25], or reflex bronchoconstriction, all due to pulmonary or bronchial vascular engorgement [21, 24–26]. In normal subjects, increased cardiac output by rapid saline infusion actuated bronchial hyperreactivity, supposedly by mechanical impingement of the airway lumen by increased mucosal thickness [27]. Also, when prohibiting deep breaths in normal subjects, loss of smooth muscle relaxation led to acute airway narrowing and bronchial hyperreactivity, implying that asthma-like symptoms can be imitated in case of impaired deep inspiration [28, 29]. In support of these findings, left-to-right shunting in ASD patients might similarly augment bronchial hyperreactivity, resulting in asthma-like symptomatology.

Whichever pathophysiological mechanism is responsible, asthma-like symptoms in ASD patients decrease significantly after closure of the defect. The possibility exists of wrongly diagnosing asthma in such patients while they should be recognised as potential ASD patients; however, as of yet we cannot differentiate between bronchial asthma and ASD patients with asthma-like symptoms. Currently the only way to obviate asthma misdiagnosis is to optimise awareness that ASD is included in the differential diagnosis of bronchial asthma, especially when bronchodilation appears insufficient. More insight into the pathophysiology of these asthma-like symptoms is required to provide an attainable tool for distinguishing ASD patients from the large pool of asthmatics. Also, further studies are needed to objectify these asthma-like symptoms, e. g. by bronchoprovocation testing.

Several limitations must be noted. This study had a retrospective design, therefore the questionnaire results relied on patients’ recollection of symptoms. Although paired testing was performed to provide for per-patient changes, as of now the Mini-AQLQ and ACQ6 questionnaires are validated in asthmatics only. Questions on dyspnoea and wheezing are acceptably sensitive and specific for detecting bronchial hyperreactivity in the general population, but patient-reported outcomes remain subjective. Statistical analysis of group differences between non-asthmatics and asthmatics could not be performed due to the small number of asthmatics in this patient cohort; therefore, only frequency comparisons were made.

## Conclusions

A high prevalence of patient-reported cough, wheezing, chest tightness and effort dyspnoea is present in patients with unrepaired ASD. Percutaneous ASD closure leads to significant symptom resolution or reduction in the majority of these patients (80 %, *p* < 0.001). The prevalence and pathophysiology of asthma-like symptoms in this patient population remains to be further investigated in prospective studies.
